# Flexible Sandwich-Shaped Cellulose Nanocrystals/Silver Nanowires/MXene Films Exhibit Efficient Electromagnetic-Shielding Interference Performance

**DOI:** 10.3390/nano14070647

**Published:** 2024-04-08

**Authors:** Shasha Yan, Ling Li, Hong Zhang, Qiubo Fu, Xingbo Ge

**Affiliations:** 1 School of Chemistry and Chemical Engineering, Southwest Petroleum University, Chengdu 610500, China; shasha_yan977@163.com (S.Y.); 13688185431@163.com (L.L.); 18483282426@sina.cn (H.Z.); 2 Institute of Chemical Materials, China Academy of Engineering Physics, Mianyang 621900, China

**Keywords:** electromagnetic interference shielding, sandwich-shaped construction, MXene, silver nanowires (AgNWs), cellulose nanocrystals (CNCs)

## Abstract

The electromagnetic pollution problem is becoming increasingly serious due to the speedy advance of electronic communication devices. There are broad application prospects for the development of flexible, wearable composite films with high electromagnetic interference (EMI)-shielding performance. The MX@AC composite films were prepared from MXene, silver nanowires (AgNWs) and cellulose nanocrystals (CNCs) with a sandwich structure. Benefiting from the upper and lower frame structure formed by winding 1D AgNWs and CNC, the tensile strength of the MX@AC was improved to 35 MPa (12.5 wt% CNC content) from 4 MPa (0 wt% CNC content). The high conductivity of MXene and AgNWs resulted in the MX@AC composite film conductivity up to 90,670 S/m, EMI SE for 90 dB, as well as SSE/t up to 7797 dB cm^2^ g^−1^. And the MX@AC composite film was tested for practical application, showing that it can effectively isolate electromagnetic waves in practical application.

## 1. Introduction

Electromagnetic pollution is increasing due to the rapid development of 5G/6G communication systems [1,2]. Thus, the development of flexible wearable composite films with high electromagnetic interference (EMI)-shielding performance can protect the human body from electromagnetic radiation on the one hand and protect the normal operation of precision equipment on the other. In addition, it can also provide technical support for the preparation of stealth materials used in aerospace or military fields [3,4]. Conventional metal materials usually have excellent EMI-shielding properties; however, they suffer from drawbacks such as easy corrosion and poor elasticity, making it difficult to utilize them in a variety of situations [5]. With the increasing demand for wearable materials, researchers are focusing their interest on EMI-shielding materials based on polymers [6,7,8]. However, polymer-based EMI-shielding materials require the introduction of large amounts of conductive material, and polymers are mostly petrochemicals, which are unsustainable resources [9,10]. Carbon materials have excellent electrical conductivity as well as good mechanical properties, such as graphene–carbon nanotubes. For example, Jia et al. [11] used carboxylic acid-modified multiwalled carbon nanotubes (c-MWNCT) with aramid nanofibers (ANF) to prepare a membrane with a thickness of 37 μm and shielding effectiveness (SE) of 19.68 dB. Li et al. [12] produced composite membranes through embedding calcium alginate (CA) molecules with embedded graphene oxide (rGO) nanosheets, achieving an SE of 25.78 dB at the film of only 12 μm of thickness. Although there have been many studies on the fabrication of EMI-shielding membranes from carbon materials, the problem of poor dispersion has been limiting their wide application [13,14,15].

A new emerging two-dimensional material, MXenes, has garnered widespread attention in recent years [16,17]. One of the most researched and reported is Ti_3_C_2_T_x_ MXene, which is extensively used to prepare EMI-shielding materials because of its great electrical conductivity, hydrophilicity as well as ease of processing [18,19,20]. Liu et al. utilized MXene combined with phase change material (PCM) to prepare composites with great EMI-shielding efficiency and effective heat management with EMI SE as high as 64.7 dB [21]. Zhou et al. combined single-walled carbon nanotubes (SWNTs) and Ti_3_C_2_T_x_ MXene to prepare a composite film with the EMI SE as high as 108.1 dB for a composite film thickness of 296 µm [22]. However, Ti_3_C_2_T_x_ MXene has the disadvantages of easy oxidation and poor mechanical properties [23,24,25]. Many researchers have combined MXene with polymers to enhance the mechanical properties of composite films [26,27]. Lan et al. alternately coated MXene and polyethyleneimine (PEI) on fabrics to prepare composite films with EMI-shielding properties and flame-retardant properties [28]. Sha et al. prepared PE@PET/MXene composite films by loading MXene nanosheets on PE@PET fabrics followed by heat pressing. The EMI SE of the composite film in the X-band was up to 50 dB [29]. Cheng et al. introduced MXene with silver nanowires (AgNWs) to polyvinylidene fluoride (PVDF) and prepared composite films exhibiting an EMI SE of 41.26 dB when the thickness was 600 μm, although the mechanical properties were enhanced to about 40 MPa [30]. This makes it hard for the requirements of ultra-thinness and high EMI-shielding performance to be met. And most polymers are non-renewable and environmentally unfriendly [31]. Therefore, one-dimensional (1D) organic nanofiber materials are used for enhancing the mechanical performance of MXene [32]. In addition, fiber nanofibers (CNF) were used as a reinforcing layer in combination with MXene to prepare a flexible composite film, which showed excellent shielding performance of 54 dB [33]. Yang et al. used an ultrafine cellulose sheet layer (UCL) to connect the MXene nanosheets, and the prepared MXCS composite film showed excellent mechanical properties and high EMI-shielding performance. The EMI SE value of MXCS can reach more than 54 dB at 2–18 GHz [34].

One-dimensional (1D) cellulose nanomaterials, which are green and sustainable, can significantly improve the mechanical performance of materials [35,36]. But cellulose additives are insulating, and as cellulose increases, it reduces the EMI-shielding performance of the composite film [37,38,39,40]. AgNWs have excellent conductivity and elevated aspect ratios, and EMI-shielding materials prepared from silver nanowires have excellent properties [32,41,42,43]. Cheng et al. fabricated composite membranes with a conductive framework using MXene, AgNWs and CNF, which have a Janus structure that ensures the high electromagnetic-shielding performance while possessing excellent mechanical properties. When the content of MXene/AgNWs is 30 wt%, the EMI SE achieves 43.65 dB [44]. Zhou et al. prepared films possessing the unique asymmetric structure with MXene/AgNWs/CNF, where the composite films were externally encapsulated by CNF and had independent MXene and AgNWs layers inside. In addition, it is proposed that due to the size mismatch between MXene and AgNWs, the films made of independent MXene and AgNWs layers have superior properties to those in the case of a MXene/AgNWs mixture. The prepared composite membranes have excellent mechanical properties with EMI SE up to 55.9 dB [45]. It is shown that concentrating the conductive layer and preparing the composite film into a three-layer frame structure improves the EMI-shielding performance of the composite film while allowing its mechanical properties to be improved as much as possible.

Hence, in this study, we designed and prepared CNC/AgNWs/MXene composite films with symmetric structures by layer-by-layer deposition using vacuum-assisted filtration. CNC was chosen mainly because of its greater rigidity, smaller diameter than CNF, and weaker agglomeration tendency [35]. By introducing AgNWs and a lower doping amount of CNC, a framework structure consisting of one-dimensional nanowires entangled in the upper and lower layers of MXene films was constructed, which led to a significant improvement in the mechanical properties of the composite films. Through concentrating the conductive materials to prepare the conductive layer, the resulting CNC/AgNWs/MXene composite film has an EMI-shielding performance of up to 90 dB. The prepared CNC/AgNWs/MXene composite films had effective application function for cell phone signal blocking. We are confident that the composite films could be widely used in electronic communications, aerospace and other fields.

## 2. Materials and Methods

### 2.1. Materials

Hydrochloric acid (HCl) was offered by Chengdu Kelong Chemical Co., Ltd., Chengdu, China, Lithium fluoride (LiF) was bought from Macklin Reagent Company. Ti_3_AlC_2_ (400 mesh) was obtained from Jilin 11 Co., Ltd., Jilin, China. Cellulose nanocrystal (CNC) was derived from Huzhou ScienceK Co., Ltd., Zhejiang, China. Silver nanowires were purchased from Nanjing XFNANO Material Technology Co., Ltd., Nanjing, China.

### 2.2. Synthesis of Ti_3_C_2_T_x_ MXene

Ti_3_C_2_T_x_ MXene was prepared by the classical in situ HF method [46]. Etching away the aluminum layer of the Ti_3_AlC_2_ MAX phase was used to obtain Ti_3_C_2_T_x_ MXene nanosheets. First, 3.2 g of LiF with 40 mL of HCl (9 M) was dissolved. Subsequently, 2 g of Ti_3_AlC_2_ was stirred in and reacted at 40 °C for 48 h. At the end of the reaction, the multilayer Ti_3_C_2_Tx MXene (m-Ti_3_AlC_2_) was obtained by repeated centrifugation with deionized water to make pH > 6. It was then ultrasonicated for 1 h and centrifuged at 3500 rpm for another 1 h to generate divested Ti_3_C_2_T_x_ MXene nanosheets (d-Ti_3_C_2_T_x_).

### 2.3. Preparation of MX@AC Composite Films

Currently, CNC was dissolved in deionized water; then, AgNWs were added and stirred for 5 hours producing a mixed AgNWs/CNC solution. The mixed solution was alternately vacuum filtered with the MXene solution to make a sandwich-shaped self-supporting composite film. Among them, the top and bottom layers of the composite film formed a symmetric structure, both of which were AgNWs/CNC, and the middle layer was MXene. The mass of MXene in all composite films was 0.102 g, and the AgNWs was 0.068 g. The ratios of the total mass of MXene and AgNWs to the CNC mass in the different composite films were 9:1, 7:1, and 5:1, respectively, and these were named MX@A9C1, MX@A7C1, and MX@A5C1, respectively. To investigate the influence of AgNWs and CNC with respect to the properties of the composite films, composite films introducing only AgNWs or CNC, named MX@A and MX@C (where the CNC content is the same as in MX@A7C1), were also prepared.

### 2.4. Characterization

The morphology of MXene, CNC, AgNWs and MX@AC composite films was deter-mined by scanning electron microscopy (SEM, Regulus 8230, Tokyo, Japan), transmission electron microscopy (TEM, JEM 2100, Tokyo, Japan), and atomic force microscopy (AFM, Bruker Dimension Icon, Saarbrucken, Germany). X-ray diffraction (XRD) was used to measure the structure of the MX@AC composite film. The mechanical properties were tested by an electronic universal testing machine (UTM4103, Shenzhen, China). The size of the samples for mechanical property testing was 4 cm * 0.5 cm. The EMI-shielding properties of composite film at 8.2 to 12.4 GHz (X-band) was tested using an Agilent PNA N5234A vector network analyzer. We measured the EMI-shielding performance of the composite films using the waveguide method. The waveguide method is used to measure the EMI-shielding performance of composite films at 8.2–12.4 GHz. The size of the composite film is required to be 22.86 mm * 10.16 mm [47]. The scattering parameters (S_11_, S_12_, S_21_, S_22_) of the dual port network were measured using the vector network analyzer, where S_ij_ represents the energy injected by port j measured on port i [48]. The power coefficients of transmittance (T), reflectance (R) and absorptance (A) can be calculated using the scattering parameter with the following formula:

(1) $$R={\left|S_{11}\right|}^{2}={\left|S_{22}\right|}^{2}$$

(2) $$T={\left|S_{12}\right|}^{2}={\left|S_{21}\right|}^{2}$$

(3) R+T+A=1

The total EMI SE (SE_T_) is the sum of reflection loss (SE_R_), absorption loss (SE_A_) and multiple internal reflections (SE_M_). SEM can be ignored when SE_T_ ≥ 10 dB [49].

(4) $${\mathrm{SE}}_{R}=-10\log(1\;-\;R)\;$$

(5) $${\mathrm{SE}}_{A}=-10\log[T/(1\;-\;R)]$$

(6) $${\mathrm{SE}}_{T}{=\mathrm{SE}}_{A}{+\mathrm{SE}}_{R}{+\mathrm{SE}}_{M}$$

In addition, the thickness of a shielding material has an effect on its EMI SE, so the absolute shielding effectiveness of a material (SSE/t) is defined as the SE value divided by the thickness and density of the sample, and the unit of SSE/t is dB cm^2^ g^−1^ [50].

## 3. Results and Discussion

### 3.1. Morphological and Structural Characterization of MX@AC Composite Films

**Figure 1:**
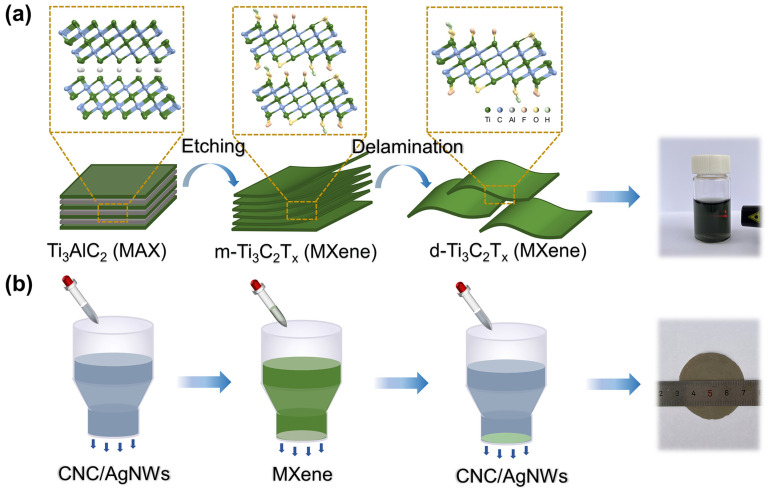
Preparation process of (**a**) Ti_3_C_2_T_x_ MXene, (**b**) MX@AC composite films.

**Figure 2:**
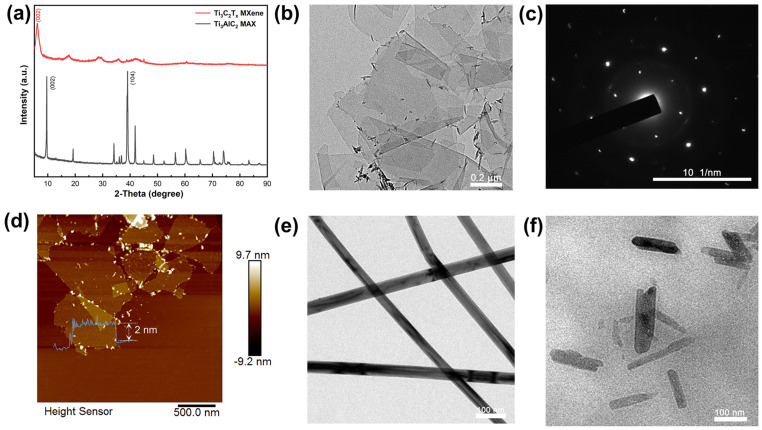
(**a**) XRD spectra of Ti_3_C_2_T_x_ MXene and Ti_3_AlC_2_ MAX. TEM (**b**,**c**) and AFM (**d**) images of Ti_3_C_2_T_x_ MXene. TEM image of (**e**) AgNWs, (**f**) CNC.

The fabrication process is illustrated in Figure 1 for MX@AC composite films. Firstly, Ti_3_AlC_2_ was etched with HCl/LiF to prepare monolayer/few-layer Ti_3_C_2_T_x_ MXene. The prepared MXene solutions exhibited a clear Tyndall effect with good dispersion. Sandwiched composite films were prepared by layer-by-layer filtration using MXene as the intermediate layer and mixed solutions of AgNWs and CNC as the upper and lower layers with a symmetric architecture, and the composite films had a diameter of 4 cm. In Figure 2a, the XRD image of MXene shows that the (002) peak of etched Ti_3_C_2_T_x_ MXene is shifted to a lower angle with respect to Ti_3_AlC_2_, which indicates that the Al atomic layer was successfully etched in Ti_3_AlC_2_ [51]. The thickness of the monolayer Ti_3_C_2_T_x_ MXene obtained by ultrasonic stripping is about 2 nm, as can be seen from the TEM and AFM of the MXene in Figure 2b,d. Figure 2c shows a high-resolution TEM image of MXene, clearly displaying the hexagonal arrangement of atoms. In Figure 2e,f, the TEM images of AgNWs and CNCs are shown, respectively. Figure 2e shows that AgNWs have a high aspect ratio, and the diameter of AgNWs can be measured to be around 40 nm.

**Figure 3:**
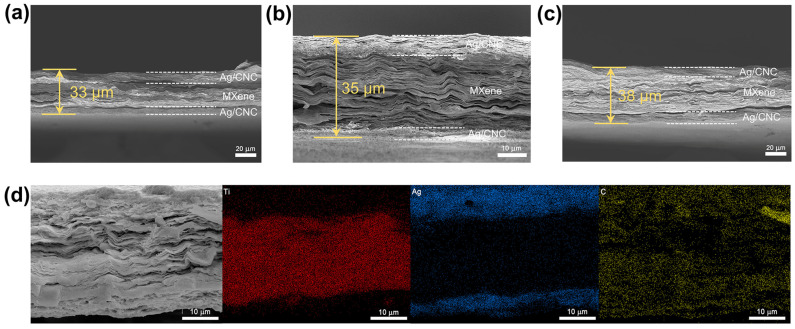
SEM images of cross-sections of (**a**) MX@A9C1, (**b**) MX@A7C1 and (**c**) MX@A5C1. (**d**) Elemental mapping of the cross-section of MX@A9C1.

Figure 3a–c demonstrate the SEM images of cross-sections of MX@AC with different CNC contents. The SEM images show that the MX@AC composite film exhibits a sandwiched shape and the middle layer, MXene, shows a regularly ordered laminate structure. There was also no delamination between each layer of the sandwich-shaped composite film. The thicknesses of the composite films MX@A9C1, MX@A7C1, and MX@A5C1 were measured to be 33 μm, 35 μm, and 38 μm by SEM images, respectively. Based on the SEM images of MX@A and MX@C from the Supplementary Information, it can be concluded that the thickness of the composite film was 32 µm and 34 µm, respectively (Figure S1a,b). The elemental mapping of the cross-section of the MX@A9C1 shows that Ti elements are dispersed only in the middle layer, while silver elements are dispersed in the top and bottom layers as envisioned in the experimental design, which indicates that the design of the sandwich structure is successful.

**Figure 4:**
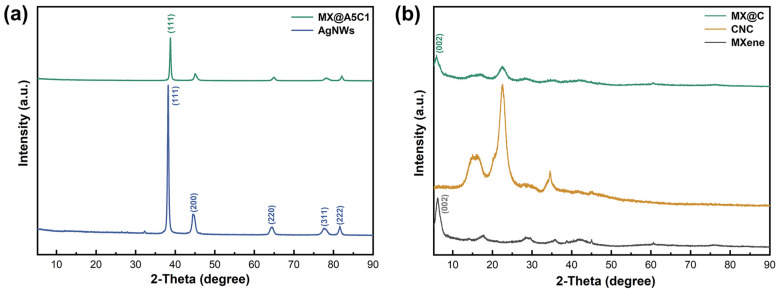
XRD patterns of (**a**) MX@A5C1 and AgNWs, (**b**) MX@C, CNC and MXene.

XRD spectra show how the lattice structure of the combined MX@AC films changes. Figure 4a illustrates the XRD pattern of MX@A5C1 and AgNWs. The diffraction peak of MX@A5C1 at 2θ = 38° is the (111) diffraction peak of AgNWs. The XRD patterns of the prepared MX@C composite films (Figure 4b) show diffraction peaks associated with MXene and CNC when the upper and lower layers of the composite films contain only CNC. In contrast, the diffraction peaks associated with CNC were not seen in MX@A5C1. The reasons for this are that the amount of CNC was doped to a lesser extent, and there was too much intensity in the diffraction peaks of AgNWs.

### 3.2. Mechanical Properties of MX@AC

**Figure 5:**
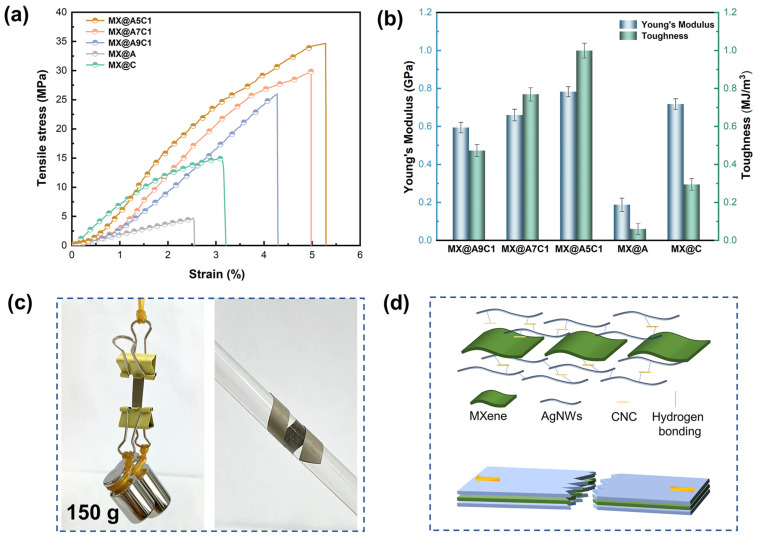
(**a**) Stress–strain curves exhibited by films. (**b**) Young’s modulus and toughness of MX@AC, MX@A and MX@C. (**c**) Optical images of MX@AC carrying 150 g weight and laminated on glass rod. (**d**) Fracture schematic of MX@AC.

Figure 5a shows the stress–strain curve of the composite films. As we expected, the more CNC content there is in the structure at the outer frame of the sandwich composite film, the better the mechanical performance of the laminated film. By increasing the CNC content in the composite film from 0 wt% to 12.5 wt%, there was an increase in the tensile strength of the composite film to 35 MPa (MX@A5C1) from 4 MPa (MX@A), and the tensile properties of the composite film were improved by a factor of 8.75 after addition of the low content of CNC. Figure 5b displays the Young’s modulus and toughness of the composite films. With the addition of CNC doping, the Young’s modulus and toughness are improved in the composite films. The Young’s modulus and toughness of MX@A are 0.19 Gpa and 0.06 MJ/m^3^, respectively, while the Young’s modulus and toughness of MX@A5C1 are 0.79 Gpa and 0.99 MJ/m^3^, respectively, being 4.16 and 16.5 times better than those of MX@A. Figure 5c shows that an MX@AC at 0.4 cm in width can sustain a weight of 150 g unbroken and has good flexibility.

From the test results of Figure 5a,b on the mechanical properties of the composite film, it can be seen that the composite film MX@C with only CNC added to the outer frame structure of the composite film shows a lower tensile strength than that of MX@A7C1. The main reason for this is that in the outer framework structure of the MX@AC composite film, the longer AgNWs and CNC are interconnected with each other through hydrogen bonding to form an entangled 1D network structure, which increases the overall mechanical performance of the composite film. During stretching of both ends of the composite film, the outer frame structure will be subjected to a larger portion of the stress of the composite film (Figure 5d). We compared the tensile strength and electromagnetic-shielding properties of the prepared MX@AC composite films with the findings of the published literature. As shown in Figure S2, the MX@AC composite film has some advantages over other research results in the literature, possessing high electromagnetic-shielding properties along with good mechanical properties. Table S2 lists the literature information cited in Figure S2.

### 3.3. EMI-Shielding Performance of MX@AC Composite Films

**Figure 6:**
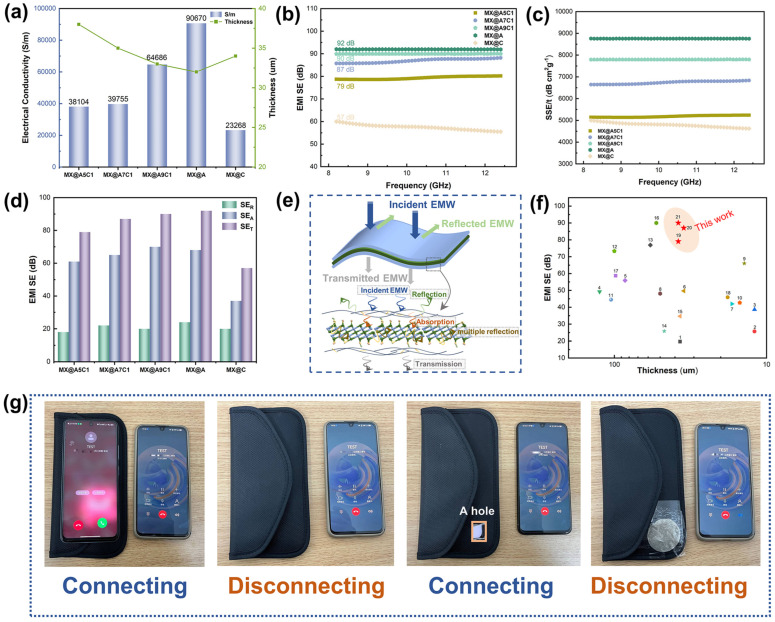
(**a**) Conductivity and thickness of MX@AC. (**b**) EMI SE of MX@AC at X-band. (**c**) SSE/t value of MX@AC composite films at X-band. (**d**) SE_Total_, SE_A_ and SE_R_ of MX@AC composite films. (**e**) Process diagram of EMI shielding by MX@AC composite film. (**f**) Thickness and EMI SE of different samples for comparison. (**g**) EMI shielding effect of MX@AC composite film in practical applications.

The conductivity of the composite films has been tested in order to investigate the EMI-shielding properties of MX@AC composite films. The conductivity of the MX@A was as high as 90,670 S/m when the top and bottom frame structures of the film contained only AgNWs, as shown in Figure 6a. Even the MX@A5C1 composite film, which contained the largest amount of CNC, had a conductivity of 38,104 S/m. Excellent conductivity is strongly correlated with the high EMI-shielding properties of the composite films. Benefiting from the sandwiched frame structure formed by winding 1D AgNWs with CNC, the MX@AC composite films have improved the mechanical properties by introducing CNC while ensuring the EMI SE is not weakened as much as possible. The MX@A9C1 can achieve an EMI SE of up to 90 dB while the thickness is only 33 µm (Figure 6b). When the composite film contained only CNC, the EMI SE value was 57 dB, which indicated that a significant improvement in the EMI-shielding performance of the composite film was achieved by the introduction of AgNWs. To further confirm the excellent shielding properties of MX@AC, we evaluated the EMI SE of the composite film with a specific EMI SE (SSE/t, defined as the product of the SE value divided by the density and thickness of the sample). The SSE/t values of different composite films were calculated, with MX@A9C1 reaching as high as 7797 dB cm^2^g^−1^, as shown in Figure 6c.

Figure 6d,e describe in detail the shielding mechanism as well as the shielding process of the MX@AC composite. When an electromagnetic wave (EMW) acts on the surface of the film, a fraction is immediately reflected, which is called microwave reflection (SE_R_). The remaining EMW interacts with AgNWs and MXene of elevated charge density, resulting in energy loss in EMW, which is called microwave absorption (SE_A_). At the same time, multiple layers of internal reflections, known as SEM, are generated inside the composite film. Since the top and bottom layers of the composite film are formed by AgNWs and CNC to form a highly conductive framework structure, it is difficult for the EMW incident into the composite film to be transmitted out, so only a small amount of the remaining EMW will be transmitted through the composite film. Where total EMI SE (SE_T_) is the sum of SE_A_, SE_R_ and SE_M_, when SE_T_ ≥ 10 dB, SE_M_ can be neglected. Figure 6d shows that both SE_R_ and SE_A_ contribute to SE_T_. Figure 6f shows a comparison of the MX@AC composite films with a composite film of a different material with respect to thickness and EMI-shielding practices. The EMI-shielding practices of MX@AC is better than most composite films at the same thickness level. Table S1 lists information about the literature cited in Figure 6e.

Moreover, MX@AC composite film was tested for practical applications. As shown in Figure 6g, when two cell phones are placed outside the signal shielding bag, the dialing cell phone shows that it is ringing, indicating that the signal can be connected. When one of the cell phones was put into the signal shielding bag, both cell phone signals were cut off, showing that they were in the process of dialing, and the phone in the shielding bag did not vibrate. A hole is then cut in the signal shielding bag, electromagnetic waves leak from the small hole, and the two phones can be connected. When the cut hole is covered by the MX@AC composite film, the two phones are disconnected, indicating that the MX@AC composite film can effectively isolate electromagnetic waves.

## 4. Conclusions

In conclusion, we prepared sandwich-shaped MX@AC composite films by a vacuum filtration device. By introducing a small amount of CNC to form a framework structure of 1D AgNWs and CNC wrapped around the top and bottom of the MXene film, the tensile strength was enhanced from 4 MPa by a factor of 8.75 (MX@A) to 35 MPa (MX@A5C1) for the composite film. The Young’s modulus and toughness of MX@AC are 4.16 and 16.5 times higher than that of MX@A, and MX@A5C1 can sustain a weight of 150 g without breaking. In addition, the prepared MX@AC composite films exhibited superior electrical conductivity and EMI-shielding properties, and the MX@AC had a conductivity up to 90,670 S/m. The EMI SE of MX@AC is 79 dB, 87 dB, and 90 dB at the thickness of 33 μm, 35 μm, and 38 μm, respectively, and the SSE/t is up to 7797 dB cm^2^ g^−1^. And MX@AC composite film has practical application value, which can effectively shield cell phone signals, and it has a broad application prospect in the field of wearable, electronic devices, artificial intelligence and so on.

## Data Availability

The data presented in this study are available on request from the corresponding author.

## Supplementary Materials

The following supporting information can be downloaded at https://www.mdpi.com/article/10.3390/nano14070647/s1, Figure S1: SEM images of cross-sections for (a) MX@A, (b) MX@C; Figure S2: Tensile stress and EMI SE of different samples for comparison; Table S1: Comparison of the EMI-shielding performance of the MX@AC composite films and other materials; Table S2: Comparison of the tensile strength of MX@AC composite film with other materials Refs. [52,53,54,55,56,57,58,59,60,61,62,63,64,65] are cited in the supplementary materials.

## References

[1] Cheng J., Li C., Xiong Y., Zhang H., Raza H., Ullah S., Wu J., Zheng G., Cao Q., Zhang D. (2022). Recent Advances in Design Strategies and Multifunctionality of Flexible Electromagnetic Interference Shielding Materials. Nano-Micro Lett..

[2] Jia X., Li Y., Shen B., Zheng W. (2022). Evaluation, fabrication and dynamic performance regulation of green EMI-shielding materials with low reflectivity: A review. Compos. Part B Eng..

[3] Liang C., Gu Z., Zhang Y., Ma Z., Qiu H., Gu J. (2021). Structural Design Strategies of Polymer Matrix Composites for Electromagnetic Interference Shielding: A Review. Nano-Micro Lett..

[4] Wang Z., Cheng Z., Fang C., Hou X., Xie L. (2020). Recent advances in MXenes composites for electromagnetic interference shielding and microwave absorption. Compos. Part A Appl. Sci. Manuf..

[5] Park J.S., Park J.Y., Lee K., Cho Y.S., Shin H., Jung Y., Park C.R., Kim T., Kim J.H., Yang S.J. (2023). Large-scalable, ultrastable thin films for electromagnetic interference shielding. J. Mater. Chem. A.

[6] He J., Han M., Wen K., Liu C., Zhang W., Liu Y., Su X., Zhang C., Liang C. (2023). Absorption-dominated electromagnetic interference shielding assembled composites based on modular design with infrared camouflage and response switching. Compos. Sci. Technol..

[7] Ma C., Ma M.G., Si C., Ji X.X., Wan P. (2021). Flexible MXene-Based Composites for Wearable Devices. Adv. Funct. Mater..

[8] Sang M., Liu G., Liu S., Wu Y., Xuan S., Wang S., Xuan S., Jiang W., Gong X. (2021). Flexible PTFE/MXene/PI soft electrothermal actuator with electromagnetic-interference shielding property. Chem. Eng. J..

[9] Zhang Y., Ruan K., Zhou K., Gu J. (2023). Controlled Distributed Ti3C2Tx Hollow Microspheres on Thermally Conductive Polyimide Composite Films for Excellent Electromagnetic Interference Shielding. Adv. Mater..

[10] Zhuo L., Cai Y., Shen D., Gou P., Wang M., Hu G., Xie F. (2023). Anti-oxidation polyimide-based hybrid foams assembled with bilayer coatings for efficient electromagnetic interference shielding. Chem. Eng. J..

[11] Jia F., Lu Z., Liu Y., Li J., Xie F., Dong J. (2022). Carboxylate-Decorated Multiwalled Carbon Nanotube/Aramid Nanofiber Film for Tunable Electromagnetic Interference Shielding Performance and Rapid Electric Heating Capacity. ACS Appl. Polym. Mater..

[12] Jia L.-C., Sun W.-J., Zhou C.-G., Yan D.-X., Zhang Q.-C., Li Z.-M. (2018). Integrated strength and toughness in graphene/calcium alginate films for highly efficient electromagnetic interference shielding. J. Mater. Chem. C.

[13] Wei Q., Pei S., Qian X., Liu H., Liu Z., Zhang W., Zhou T., Zhang Z., Zhang X., Cheng H.M. (2020). Superhigh Electromagnetic Interference Shielding of Ultrathin Aligned Pristine Graphene Nanosheets Film. Adv. Mater..

[14] Wu Z., Dong J., Li X., Zhao X., Ji C., Zhang Q. (2022). Interlayer decoration of expanded graphite by polyimide resins for preparing highly thermally conductive composites with superior electromagnetic shielding performance. Carbon.

[15] Zhang Y., Ruan K., Shi X., Qiu H., Pan Y., Yan Y., Gu J. (2021). Ti3C2Tx/rGO porous composite films with superior electromagnetic interference shielding performances. Carbon.

[16] Cao M.-S., Cai Y.-Z., He P., Shu J.-C., Cao W.-Q., Yuan J. (2019). 2D MXenes: Electromagnetic property for microwave absorption and electromagnetic interference shielding. Chem. Eng. J..

[17] Iqbal A., Sambyal P., Koo C.M. (2020). 2D MXenes for Electromagnetic Shielding: A Review. Adv. Funct. Mater..

[18] Liu J., Liu Z., Zhang H.B., Chen W., Zhao Z., Wang Q.W., Yu Z.Z. (2019). Ultrastrong and Highly Conductive MXene-Based Films for High-Performance Electromagnetic Interference Shielding. Adv. Electron. Mater..

[19] Wang J.Q., Qian P.F., Lou T.J., Wang W., Geng W.H., Jing L.C., Bao Z.L., Wang T., Geng H.Z. (2021). Vacuum-Assisted Layer-by-Layer Carbon Nanotube/Ti3C2TX MXene Films for Detecting Human Movements. Adv. Mater. Technol..

[20] Qian K., Wu H., Fang J., Yang Y., Miao M., Cao S., Shi L., Feng X. (2021). Yarn-ball-shaped CNF/MWCNT microspheres intercalating Ti_3_C_2_T_x_ MXene for electromagnetic interference shielding films. Carbohydr. Polym..

[21] Liu H., Fu R., Su X., Wu B., Wang H., Xu Y., Liu X. (2021). MXene confined in shape-stabilized phase change material combining enhanced electromagnetic interference shielding and thermal management capability. Compos. Sci. Technol..

[22] Zhou B., Li Y., Li Z., Ma J., Zhou K., Liu C., Shen C., Feng Y. (2021). Fire/heat-resistant, anti-corrosion and folding Ti_2_C_3_T_x_ MXene/single-walled carbon nanotube films for extreme-environmental EMI shielding and solar-thermal conversion applications. J. Mater. Chem. C.

[23] Seredych M., Shuck C.E., Pinto D., Alhabeb M., Precetti E., Deysher G., Anasori B., Kurra N., Gogotsi Y. (2019). High-Temperature Behavior and Surface Chemistry of Carbide MXenes Studied by Thermal Analysis. Chem. Mater..

[24] Li X., Yang M., Qin W., Gu C., Feng L., Tian Z., Qiao H., Chen J., Chen J., Yin S. (2023). MXene-based multilayered flexible strain sensor integrating electromagnetic shielding and Joule heat. Colloids Surf. A Physicochem. Eng. Asp..

[25] Lee Y., Kim S.J., Kim Y.-J., Lim Y., Chae Y., Lee B.-J., Kim Y.-T., Han H., Gogotsi Y., Ahn C.W. (2020). Oxidation-resistant titanium carbide MXene films. J. Mater. Chem. A.

[26] Xu D., Huang Q., Yang L., Chen Y., Lu Z., Liu H., Han P., Guo L., Wang C., Liu C. (2023). Experimental design of composite films with thermal management and electromagnetic shielding properties based on polyethylene glycol and MXene. Carbon.

[27] Zhang Y., Gao Q., Sheng X., Zhang S., Chen J., Ma Y., Qin J., Zhao Y., Shi X., Zhang G. (2023). Flexible, robust, sandwich structure polyimide composite film with alternative MXene and Ag NWs layers for electromagnetic interference shielding. J. Mater. Sci. Technol..

[28] Lan C., Jia H., Qiu M., Fu S. (2021). Ultrathin MXene/Polymer Coatings with an Alternating Structure on Fabrics for Enhanced Electromagnetic Interference Shielding and Fire-Resistant Protective Performances. ACS Appl. Mater. Interfaces.

[29] Sha Z., He H., Ma H., Hong B., Lu J., Fei X., Zhu M. (2024). All-in-one integrated flexible PE@PET/MXene films for high-performance electromagnetic shields with self-reinforced conductivity and mechanical properties. Carbon.

[30] Cheng H., Pan Y., Chen Q., Che R., Zheng G., Liu C., Shen C., Liu X. (2021). Ultrathin flexible poly(vinylidene fluoride)/MXene/silver nanowire film with outstanding specific EMI shielding and high heat dissipation. Adv. Compos. Hybrid Mater..

[31] Zhang W., Ji X.-X., Ma M.-G. (2023). Emerging MXene/cellulose composites: Design strategies and diverse applications. Chem. Eng. J..

[32] Ma Z., Kang S., Ma J., Shao L., Zhang Y., Liu C., Wei A., Xiang X., Wei L., Gu J. (2020). Ultraflexible and Mechanically Strong Double-Layered Aramid Nanofiber–Ti_3_C_2_T_x_ MXene/Silver Nanowire Nanocomposite Papers for High-Performance Electromagnetic Interference Shielding. ACS Nano.

[33] Ma C., Mai T., Wang P.-L., Guo W.-Y., Ma M.-G. (2023). Flexible MXene/Nanocellulose Composite Aerogel Film with Cellular Structure for Electromagnetic Interference Shielding and Photothermal Conversion. ACS Appl. Mater. Interfaces.

[34] Yang Y., Chen K., Dang B., Wang C., Chen Y., Liu M., Li Y., Zhang X., Sun Q. (2023). Flexible and mechanically strong MXene/cellulose-lamellae sheets for electromagnetic interference shielding. Chem. Eng. J..

[35] Wu N., Li B., Pan F., Zhang R., Liu J., Zeng Z. (2023). Ultrafine cellulose nanocrystal-reinforced MXene biomimetic composites for multifunctional electromagnetic interference shielding. Sci. China Mater..

[36] Cao W.-T., Chen F.-F., Zhu Y.-J., Zhang Y.-G., Jiang Y.-Y., Ma M.-G., Chen F. (2018). Binary Strengthening and Toughening of MXene/Cellulose Nanofiber Composite Paper with Nacre-Inspired Structure and Superior Electromagnetic Interference Shielding Properties. ACS Nano.

[37] Zeng Z., Wang C., Siqueira G., Han D., Huch A., Abdolhosseinzadeh S., Heier J., Nüesch F., Zhang C., Nyström G. (2020). Nanocellulose-MXene Biomimetic Aerogels with Orientation-Tunable Electromagnetic Interference Shielding Performance. Adv. Sci..

[38] Ma M., Liao X., Chu Q., Chen S., Shi Y., He H., Wang X. (2022). Construction of gradient conductivity cellulose nanofiber/MXene composites with efficient electromagnetic interference shielding and excellent mechanical properties. Compos. Sci. Technol..

[39] Guo Z., Ren P., Lu Z., Hui K., Yang J., Zhang Z., Chen Z., Jin Y., Ren F. (2022). Multifunctional CoFe_2_O_4_@MXene-AgNWs/Cellulose Nanofiber Composite Films with Asymmetric Layered Architecture for High-Efficiency Electromagnetic Interference Shielding and Remarkable Thermal Management Capability. ACS Appl. Mater. Interfaces.

[40] Liu K., Du H., Liu W., Zhang M., Wang Y., Liu H., Zhang X., Xu T., Si C. (2022). Strong, flexible, and highly conductive cellulose nanofibril/PEDOT:PSS/MXene nanocomposite films for efficient electromagnetic interference shielding. Nanoscale.

[41] Chen W., Liu L.-X., Zhang H.-B., Yu Z.-Z. (2020). Flexible, Transparent, and Conductive Ti_3_C_2_T_x_ MXene–Silver Nanowire Films with Smart Acoustic Sensitivity for High-Performance Electromagnetic Interference Shielding. ACS Nano.

[42] Tang H., Feng H., Wang H., Wan X., Liang J., Chen Y. (2019). Highly Conducting MXene–Silver Nanowire Transparent Electrodes for Flexible Organic Solar Cells. ACS Appl. Mater. Interfaces.

[43] Shi Y., Xiang Z., Cai L., Pan F., Dong Y., Zhu X., Cheng J., Jiang H., Lu W. (2022). Multi-interface Assembled N-Doped MXene/HCFG/AgNW Films for Wearable Electromagnetic Shielding Devices with Multimodal Energy Conversion and Healthcare Monitoring Performances. ACS Nano.

[44] Cheng R., Wang B., Zeng J., Li J., Xu J., Gao W., Chen K. (2023). Janus-inspired flexible cellulose nanofiber-assisted MXene/Silver nanowire papers with fascinating mechanical properties for efficient electromagnetic interference shielding. Carbon.

[45] Zhou B., Li Q., Xu P., Feng Y., Ma J., Liu C., Shen C. (2021). An asymmetric sandwich structural cellulose-based film with self-supported MXene and AgNW layers for flexible electromagnetic interference shielding and thermal management. Nanoscale.

[46] Wei Y., Zhang P., Soomro R.A., Zhu Q., Xu B. (2021). Advances in the Synthesis of 2D MXenes. Adv. Mater..

[47] Yan S., Zhang H., Li L., Fu Q., Ge X. (2024). Flexible and Recyclable MXene Nanosheet/Ag Nanowire/Cellulose Nanocrystal Composite Films for Electromagnetic Interference Shielding. ACS Appl. Nano Mater..

[48] Li Y., Xue B., Yang S., Cheng Z., Xie L., Zheng Q. (2021). Flexible multilayered films consisting of alternating nanofibrillated cellulose/Fe3O4 and carbon nanotube/polyethylene oxide layers for electromagnetic interference shielding. Chem. Eng. J..

[49] Wang Y., Peng H.-K., Li T.-T., Shiu B.-C., Zhang X., Lou C.-W., Lin J.-H. (2020). Layer-by-layer assembly of low-temperature in-situ polymerized pyrrole coated nanofiber membrane for high-efficiency electromagnetic interference shielding. Prog. Org. Coat..

[50] Fan M., Li S., Wu L., Li L., Qu M., Nie J., Zhang R., Tang P., Bin Y. (2022). Natural rubber toughened carbon nanotube buckypaper and its multifunctionality in electromagnetic interference shielding, thermal conductivity, Joule heating and triboelectric nanogenerators. Chem. Eng. J..

[51] Zhang Q., Cui J., Zhao S., Zhang G., Gao A., Yan Y. (2023). Development of Electromagnetic-Wave-Shielding Polyvinylidene Fluoride–Ti_3_C_2_T_x_ MXene–Carbon Nanotube Composites by Improving Impedance Matching and Conductivity. Nanomaterials.

[52] Liu Y., Zhang B., Wang Q., Yu G., Liu W., Bai X., Dong L. (2023). Thin, Flexible, and High-Strength Graphene Films Modified with CoFe2O4 Nanoparticle–Carbon Nanotubes Composites for Electromagnetic Interference Shielding. ACS Appl. Nano Materials.

[53] Miao M., Liu R., Thaiboonrod S., Shi L., Cao S., Zhang J., Fang J., Feng X. (2020). Silver nanowires intercalating Ti3C2Tx MXene composite films with excellent flexibility for electromagnetic interference shielding. J. Mater. Chem. C.

[54] Cui C., Xiang C., Geng L., Lai X., Guo R., Zhang Y., Xiao H., Lan J., Lin S., Jiang S. (2019). Flexible and ultrathin electrospun regenerate cellulose nanofibers and d-Ti3C2Tx (MXene) composite film for electromagnetic interference shielding. J. Alloys Compd..

[55] Liu F., Li Y., Hao S., Cheng Y., Zhan Y., Zhang C., Meng Y., Xie Q., Xia H. (2020). Well-aligned MXene/chitosan films with humidity response for high-performance electromagnetic interference shielding. Carbohydr. Polym..

[56] Ji H., Zhao R., Zhang N., Jin C., Lu X., Wang C. (2018). Lightweight and flexible electrospun polymer nanofiber/metal nanoparticle hybrid membrane for high-performance electromagnetic interference shielding. NPG Asia Mater..

[57] Qian J., Zhang Z.M., Bao R.Y., Liu Z.Y., Yang M.B., Yang W. (2020). Lightweight poly (vinylidene fluoride)/silver nanowires hybrid membrane with different conductive network structure for electromagnetic interference shielding. Polym. Compos..

[58] Yang S., Wang Y.-Y., Song Y.-N., Jia L.-C., Zhong G.-J., Xu L., Yan D.-X., Lei J., Li Z.-M. (2021). Ultrathin, flexible and sandwich-structured PHBV/silver nanowire films for high-efficiency electromagnetic interference shielding. J. Mater. Chem. C.

[59] Zhang Y., Wang L., Zhang J., Song P., Xiao Z., Liang C., Qiu H., Kong J., Gu J. (2019). Fabrication and investigation on the ultra-thin and flexible Ti3C2Tx/co-doped polyaniline electromagnetic interference shielding composite films. Compos. Sci. Technol..

[60] Wang Y., Liu R., Zhang J., Miao M., Feng X. (2021). Vulcanization of Ti3C2T MXene/natural rubber composite films for enhanced electromagnetic interference shielding. Appl. Surf. Sci..

[61] Ren W., Zhu H., Yang Y., Chen Y., Duan H., Zhao G., Liu Y. (2020). Flexible and robust silver coated non-woven fabric reinforced waterborne polyurethane films for ultra-efficient electromagnetic shielding. Compos. Part B Eng..

[62] Liao S.-Y., Wang X.-Y., Li X.-M., Wan Y.-J., Zhao T., Hu Y.-G., Zhu P.-L., Sun R., Wong C.-P. (2021). Flexible liquid metal/cellulose nanofiber composites film with excellent thermal reliability for highly efficient and broadband EMI shielding. Chem. Eng. J..

[63] Fan M., Song J., Qu M., Li S., Chen R., Ma Y., Tang P., Yuezhen B. (2023). Bacterial-Cellulose-Reinforced Graphite Nanoplate Films for Electromagnetic Interference Shielding, Heat Conduction, and Joule Heating. ACS Appl. Nano Mater..

[64] Wang T., Kong W.-W., Yu W.-C., Gao J.-F., Dai K., Yan D.-X., Li Z.-M. (2021). A Healable and Mechanically Enhanced Composite with Segregated Conductive Network Structure for High-Efficient Electromagnetic Interference Shielding. Nano-Micro Lett..

[65] Zhao W., Zhao B., Wu Z., Pei K., Qian Y., Luo K., Xu C., Liu M., Wang M., Zhang J. (2023). Dopant Engineering of Flexible MNPs/TPU/PPy Core–Shell Films for Controllable Electromagnetic Interference Shielding. ACS Appl. Mater. Interfaces.

