# Supplementary material for: Flexible Sandwich-Shaped Cellulose Nanocrystals/Silver Nanowires/MXene Films Exhibit Efficient Electromagnetic-Shielding Interference Performance
Source: Nanomaterials (Basel). 2024 Apr 8;14(7):647. doi: 10.3390/nano14070647 (PMC11013409; doi:10.3390/nano14070647)
Supplement: Supplementary file 1 [file nanomaterials-14-00647-s001.zip › nanomaterials-2934532-supplementary.pdf]

nanomaterials

MDPI

Article

Flexible Sandwich-Shaped Cellulose Nanocrystals/Silver Nanowires/MXene Films Exhibit Efficient Electromagnetic-Shielding Interference Performance

Shasha Yan [1,2], Ling Li [1], Hong Zhang [1,2], Qiubo Fu [2,*] and Xingbo Ge [1,*]

1 School of Chemistry and Chemical Engineering, Southwest Petroleum University, Chengdu 610500, China;
shasha_yan977@163.com (S.Y.); 13688185431@163.com (L.L.); 18483282426@sina.cn (H.Z.)
2 Institute of Chemical Materials, China Academy of Engineering Physics, Mianyang 621900, China
* Correspondence: fuqiubo@caep.cn (Q.F.); xbge@swpu.edu.cn (X.G.)

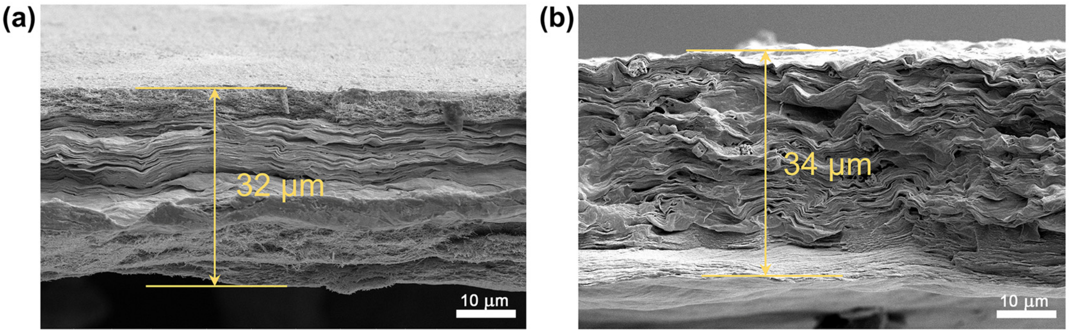

Figure S1. SEM images of cross sections for (**a**) MX@A, (**b**) MX@C.

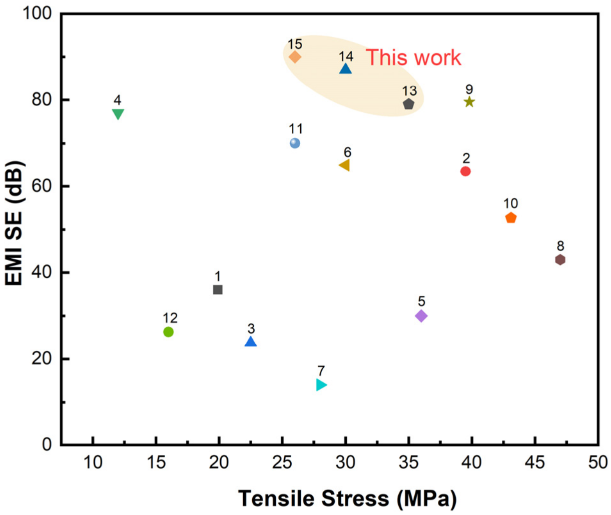

Figure S2. Tensile stress and EMI SE of different samples for comparison

Table S1. Comparison of the EMI shielding performance of the MX@AC composite films and other materials.

Sample	Materials	SE (dB)	Thickness (μm)	Ref.
1	c-MWCNTs/ANF	19.68	37	[1]
2	CA/rGO	25.7	12	[2]
3	$CoFe_2O_4$/CNTs/RGO	38.7	12	[3]
4	$Ti_3C_2T_x$ MXene/AgNWs	49.2	125	[4]
5	MXene/AgNWs/CNF	55.9	85	[5]
6	MXene/aramid nanofiber	49.7	35	[6]
7	AgNWs/MXene/NC	42	16.9	[7]
8	ANF/MXene/AgNW	48.1	50	[8]
9	MXene/CNC	66	14	[9]
10	MXene/r-CNF	42.7	15	[10]
11	CNF/MXene	44.5	105	[11]
12	CoFe2O4/MXene/CNF	73.3	100	[12]
13	CNF/PEDOT:PSS/MXene	76.99	58	[13]
14	MXene/CNF	25.8	47	[14]
15	chitosan /MXene	34.7	37	[15]
16	CPAN NF/Ag nanoparticle	90	53	[16]
17	PVDF/AgNW	58.7	98	[17]
18	PHBV/AgNW	45.9	18	[18]
19	MX@A5C1	79	38	This work
20	MX@A7C1	87	35	This work
21	MX@A9C1	90	38	This work

Table S2. Comparison of the tensile strength of MX@AC composite film with other materials

Sample	Materials	SE (dB)	tensile strength (MPa)	Ref.
1	Ti3C2Tx/c-PANI	36	19.9	[19]
2	Ti3C2Tx MXene/ NR	63.5	39.5	[20]
3	PAP@PPy	23.8	22.5	[21]
4	Ag/NWF/WPU	77	12	[22]
5	NFC/Fe3O4&CNT/PEO	30	36	[23]
6	LM/CNF	65	30	[24]
7	CNT/NR	14	28	[25]
8	BC-GNP	43	47	[26]
9	WPU/AgNWs	79.5	39.8	[27]
10	CNT/GO/PU	52.7	43.1	[28]
11	MXene/AgNWs/CNC	70	26	[29]
12	MNPs/TPU/PPy	26.3	16	[30]
13	MX@A5C1	79	35	This work
14	MX@A7C1	87	30	This work
15	MX@A9C1	90	26	This work

References

1. Jia, F.; Lu, Z.; Liu, Y.; Li, J.; Xie, F.; Dong, J. Carboxylate-Decorated Multiwalled Carbon Nanotube/Aramid Nanofiber Film for Tunable Electromagnetic Interference Shielding Performance and Rapid Electric Heating Capacity. *ACS Applied Polymer Materials* **2022**, *4*, 6342-6353, doi:10.1021/acsapm.2c00570.
2. Jia, L.-C.; Sun, W.-J.; Zhou, C.-G.; Yan, D.-X.; Zhang, Q.-C.; Li, Z.-M. Integrated strength and toughness in graphene/calcium alginate films for highly efficient electromagnetic interference shielding. *Journal of Materials Chemistry C* **2018**, *6*, 9166-9174, doi:10.1039/c8tc03151g.
3. Liu, Y.; Zhang, B.; Wang, Q.; Yu, G.; Liu, W.; Bai, X.; Dong, L. Thin, Flexible, and High-Strength Graphene Films Modified with CoFe2O4 Nanoparticle–Carbon Nanotubes Composites for Electromagnetic Interference Shielding. *ACS Applied Nano Materials* **2023**, *6*, 17031-17039, doi:10.1021/acsanm.3c03055.
4. Chen, W.; Liu, L.-X.; Zhang, H.-B.; Yu, Z.-Z. Flexible, Transparent, and Conductive Ti3C2Tx MXene–Silver Nanowire Films with Smart Acoustic Sensitivity for High-Performance Electromagnetic Interference Shielding. *ACS Nano* **2020**, *14*, 16643-16653, doi:10.1021/acsnano.0c01635.
5. Zhou, B.; Li, Q.; Xu, P.; Feng, Y.; Ma, J.; Liu, C.; Shen, C. An asymmetric sandwich structural cellulose-based film with self-supported MXene and AgNW layers for flexible electromagnetic interference shielding and thermal management. *Nanoscale* **2021**, *13*, 2378-2388, doi:10.1039/d0nr07840a.
6. Cao, W.T.; Chen, F.F.; Zhu, Y.J.; Zhang, Y.G.; Jiang, Y.Y.; Ma, M.G.; Chen, F. Binary Strengthening and Toughening of MXene/Cellulose Nanofiber Composite Paper with Nacre-Inspired Structure and Superior Electromagnetic Interference Shielding Properties. *ACS Nano* **2018**, *12*, 4583-4593, doi:10.1021/acsnano.8b00997.
7. Miao, M.; Liu, R.; Thaiboonrod, S.; Shi, L.; Cao, S.; Zhang, J.; Fang, J.; Feng, X. Silver nanowires intercalating Ti3C2Tx MXene composite films with excellent flexibility for electromagnetic interference shielding. *Journal of Materials Chemistry C* **2020**, *8*, 3120-3126, doi:10.1039/c9tc06361g.
8. Ma, Z.; Kang, S.; Ma, J.; Shao, L.; Zhang, Y.; Liu, C.; Wei, A.; Xiang, X.; Wei, L.; Gu, J. Ultraflexible and Mechanically Strong Double-Layered Aramid Nanofiber-Ti(3)C(2)T(x) MXene/Silver Nanowire Nanocomposite Papers for High-Performance Electromagnetic Interference Shielding. *ACS Nano* **2020**, *14*, 8368-8382, doi:10.1021/acsnano.0c02401.
9. Wu, N.; Li, B.; Pan, F.; Zhang, R.; Liu, J.; Zeng, Z. Ultrafine cellulose nanocrystal-reinforced MXene biomimetic composites for multifunctional electromagnetic interference shielding. *Science China Materials* **2023**, *66*, 1597-1606, doi:10.1007/s40843-022-2279-3.
10. Cui, C.; Xiang, C.; Geng, L.; Lai, X.; Guo, R.; Zhang, Y.; Xiao, H.; Lan, J.; Lin, S.; Jiang, S. Flexible and ultrathin electrospun regenerate cellulose nanofibers and d-Ti3C2Tx (MXene) composite film for electromagnetic interference shielding. *Journal of Alloys and Compounds* **2019**, *788*, 1246-1255, doi:10.1016/j.jallcom.2019.02.294.
11. Ma, M.; Liao, X.; Chu, Q.; Chen, S.; Shi, Y.; He, H.; Wang, X. Construction of gradient conductivity cellulose nanofiber/MXene composites with efficient electromagnetic interference shielding and excellent mechanical properties. *Composites Science and Technology* **2022**, *226*, 109540, doi:10.1016/j.compscitech.2022.109540.
12. Guo, Z.; Ren, P.; Lu, Z.; Hui, K.; Yang, J.; Zhang, Z.; Chen, Z.; Jin, Y.; Ren, F. Multifunctional CoFe2O4@MXene-AgNWs/Cellulose Nanofiber Composite Films with Asymmetric Layered Architecture for High-Efficiency Electromagnetic Interference Shielding and Remarkable Thermal Management Capability. *ACS Applied Materials & Interfaces* **2022**, *14*, 41468-41480, doi:10.1021/acsami.2c12555.
13. Liu, K.; Du, H.; Liu, W.; Zhang, M.; Wang, Y.; Liu, H.; Zhang, X.; Xu, T.; Si, C. Strong, flexible, and highly conductive cellulose nanofibril/PEDOT:PSS/MXene nanocomposite films for efficient electromagnetic interference shielding. *Nanoscale* **2022**, *14*, 14902-14912, doi:10.1039/d2nr00468b.

14. Ma, C.; Mai, T.; Wang, P.-L.; Guo, W.-Y.; Ma, M.-G. Flexible MXene/Nanocellulose Composite Aerogel Film with Cellular Structure for Electromagnetic Interference Shielding and Photothermal Conversion. *ACS Applied Materials & Interfaces* **2023**, *15*, 47425-47433, doi:10.1021/acsami.3c12171.
15. Liu, F.; Li, Y.; Hao, S.; Cheng, Y.; Zhan, Y.; Zhang, C.; Meng, Y.; Xie, Q.; Xia, H. Well-aligned MXene/chitosan films with humidity response for high-performance electromagnetic interference shielding. *Carbohydr Polym* **2020**, *243*, 116467, doi:10.1016/j.carbpol.2020.116467.
16. Ji, H.; Zhao, R.; Zhang, N.; Jin, C.; Lu, X.; Wang, C. Lightweight and flexible electrospun polymer nanofiber/metal nanoparticle hybrid membrane for high-performance electromagnetic interference shielding. *NPG Asia Materials* **2018**, *10*, 749-760, doi:10.1038/s41427-018-0070-1.
17. Qian, J.; Zhang, Z.M.; Bao, R.Y.; Liu, Z.Y.; Yang, M.B.; Yang, W. Lightweight poly (vinylidene fluoride)/silver nanowires hybrid membrane with different conductive network structure for electromagnetic interference shielding. *Polymer Composites* **2020**, *42*, 522-531, doi:10.1002/pc.25844.
18. Yang, S.; Wang, Y.-Y.; Song, Y.-N.; Jia, L.-C.; Zhong, G.-J.; Xu, L.; Yan, D.-X.; Lei, J.; Li, Z.-M. Ultrathin, flexible and sandwich-structured PHBV/silver nanowire films for high-efficiency electromagnetic interference shielding. *Journal of Materials Chemistry C* **2021**, *9*, 3307-3315, doi:10.1039/d0tc05266c.
19. Zhang, Y.; Wang, L.; Zhang, J.; Song, P.; Xiao, Z.; Liang, C.; Qiu, H.; Kong, J.; Gu, J. Fabrication and investigation on the ultra-thin and flexible Ti3C2Tx/co-doped polyaniline electromagnetic interference shielding composite films. *Composites Science and Technology* **2019**, *183*, 107833, doi:10.1016/j.compscitech.2019.107833.
20. Wang, Y.; Liu, R.; Zhang, J.; Miao, M.; Feng, X. Vulcanization of Ti3C2T MXene/natural rubber composite films for enhanced electromagnetic interference shielding. *Applied Surface Science* **2021**, *546*, 149143, doi:10.1016/j.apsusc.2021.149143.
21. Wang, Y.; Peng, H.-K.; Li, T.-T.; Shiu, B.-C.; Zhang, X.; Lou, C.-W.; Lin, J.-H. Layer-by-layer assembly of low-temperature in-situ polymerized pyrrole coated nanofiber membrane for high-efficiency electromagnetic interference shielding. *Progress in Organic Coatings* **2020**, *147*, 105861, doi:10.1016/j.porgcoat.2020.105861.
22. Ren, W.; Zhu, H.; Yang, Y.; Chen, Y.; Duan, H.; Zhao, G.; Liu, Y. Flexible and robust silver coated non-woven fabric reinforced waterborne polyurethane films for ultra-efficient electromagnetic shielding. *Composites Part B: Engineering* **2020**, *184*, 107745, doi:10.1016/j.compositesb.2020.107745.
23. Li, Y.; Xue, B.; Yang, S.; Cheng, Z.; Xie, L.; Zheng, Q. Flexible multilayered films consisting of alternating nanofibrillated cellulose/Fe3O4 and carbon nanotube/polyethylene oxide layers for electromagnetic interference shielding. *Chemical Engineering Journal* **2021**, *410*, 128356, doi:10.1016/j.cej.2020.128356.
24. Liao, S.-Y.; Wang, X.-Y.; Li, X.-M.; Wan, Y.-J.; Zhao, T.; Hu, Y.-G.; Zhu, P.-L.; Sun, R.; Wong, C.-P. Flexible liquid metal/cellulose nanofiber composites film with excellent thermal reliability for highly efficient and broadband EMI shielding. *Chemical Engineering Journal* **2021**, *422*, 129962, doi:10.1016/j.cej.2021.129962.
25. Fan, M.; Li, S.; Wu, L.; Li, L.; Qu, M.; Nie, J.; Zhang, R.; Tang, P.; Bin, Y. Natural rubber toughened carbon nanotube buckypaper and its multifunctionality in electromagnetic interference shielding, thermal conductivity, Joule heating and triboelectric nanogenerators. *Chemical Engineering Journal* **2022**, *433*, 133499, doi:10.1016/j.cej.2021.133499.
26. Fan, M.; Song, J.; Qu, M.; Li, S.; Chen, R.; Ma, Y.; Tang, P.; Yuezhen, B. Bacterial-Cellulose-Reinforced Graphite Nanoplate Films for Electromagnetic Interference Shielding, Heat Conduction, and Joule Heating. *ACS Applied Nano Materials* **2023**, *6*, 10202-10212, doi:10.1021/acsanm.3c01089.
27. Zhang, Y.; Gao, Q.; Sheng, X.; Zhang, S.; Chen, J.; Ma, Y.; Qin, J.; Zhao, Y.; Shi, X.; Zhang, G. Flexible, robust, sandwich structure polyimide composite film with alternative MXene and Ag NWs layers for electromagnetic interference shielding. *Journal of Materials Science & Technology* **2023**, *159*, 194-203, doi:10.1016/j.jmst.2022.10.091.

28. Wang, T.; Kong, W.-W.; Yu, W.-C.; Gao, J.-F.; Dai, K.; Yan, D.-X.; Li, Z.-M. A Healable and Mechanically Enhanced Composite with Segregated Conductive Network Structure for High-Efficient Electromagnetic Interference Shielding. *Nano-Micro Letters* **2021**, *13*, 162, doi:10.1007/s40820-021-00693-5.
29. Yan, S.; Zhang, H.; Li, L.; Fu, Q.; Ge, X. Flexible and Recyclable MXene Nanosheet/Ag Nanowire/Cellulose Nanocrystal Composite Films for Electromagnetic Interference Shielding. *ACS Applied Nano Materials* **2024**, *7*, 2702-2710, doi:10.1021/acsanm.3c04862.
30. Zhao, W.; Zhao, B.; Wu, Z.; Pei, K.; Qian, Y.; Luo, K.; Xu, C.; Liu, M.; Wang, M.; Zhang, J.; et al. Dopant Engineering of Flexible MNPs/TPU/PPy Core–Shell Films for Controllable Electromagnetic Interference Shielding. *ACS Applied Materials & Interfaces* **2023**, *15*, 28410-28420, doi:10.1021/acsami.3c02454.
